# The *fushi tarazu* zebra element is not required for *Drosophila* viability or fertility

**DOI:** 10.1093/g3journal/jkab300

**Published:** 2021-08-26

**Authors:** Patricia L Graham, Matthew D Fischer, Abhigya Giri, Leslie Pick

**Affiliations:** 1 Department of Entomology, University of Maryland, College Park, MD 20742, USA; 2 Graduate Program in Molecular & Cell Biology, University of Maryland, College Park, MD 20742, USA

**Keywords:** *Drosophila*, *cis*-regulatory elements, pair-rule gene, ftz, genome editing

## Abstract

Expression of genes in precisely controlled spatiotemporal patterns is essential for embryonic development. Much of our understanding of mechanisms regulating gene expression comes from the study of *cis*-regulatory elements (CREs) that direct expression of reporter genes in transgenic organisms. This reporter-transgene approach identifies genomic regions sufficient to drive expression but fails to provide information about quantitative and qualitative contributions to endogenous expression, although such conclusions are often inferred. Here we evaluated the endogenous function of a classic *Drosophila* CRE, the *fushi tarazu* (*ftz*) zebra element. *ftz* is a pair-rule segmentation gene expressed in seven stripes during embryogenesis, necessary for formation of alternate body segments. Reporter transgenes identified the promoter-proximal zebra element as a major driver of the seven *ftz* stripes. We generated a precise genomic deletion of the zebra element (*ftzΔZ)* to assess its role in the context of native chromatin and neighboring CREs, expecting large decreases in *ftz* seven-stripe expression. However, significant reduction in expression was found for only one stripe, *ftz* stripe 4, expressed at ∼25% of wild type levels in *ftzΔZ* homozygotes. Defects in corresponding regions of *ftzΔZ* mutants suggest this level of expression borders the threshold required to promote morphological segmentation. Further, we established true-breeding lines of homozygous *ftzΔZ* flies, demonstrating that the body segments missing in the mutants are not required for viability or fertility. These results highlight the different types of conclusions drawn from different experimental designs and emphasize the importance of examining transcriptional regulatory mechanisms in the context of the native genomic environment.

## Introduction

Precise control of gene expression by *cis*-regulatory elements (CREs) is critical to all aspects of embryonic development and organismal function (reviewed in Schaffner 2015). CRE identification is often enabled by vectors derived from naturally occurring transposable-elements that allow for integration into the genome. Reporter genes, in which candidate CREs are placed upstream of a basal promoter and the coding region of an innocuous gene such as *lacZ* or *gfp*, allow for analysis of CREs *in vivo*, in the context of a developing organism, rather than *in vitro*, cell culture or heterologous systems. These approaches have been used extensively in so-called “promoter bashing” or “enhancer bashing” experiments in many model systems, including *Drosophila melanogaster*, where P element-mediated transformation enabled the identification of many cell type-specific CREs (Rubin and Spradling 1982, 1983).

**Figure 1:**
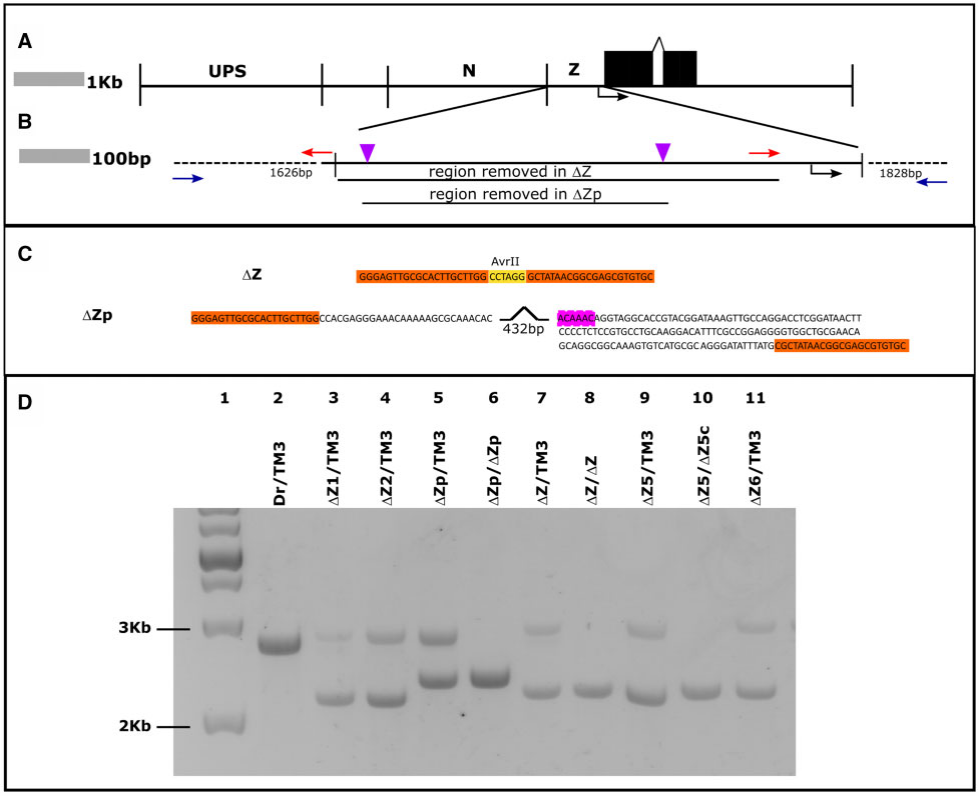
CRISPR deletion of the *ftz* zebra element. (A) Map of the *ftz* genomic region spanning the 10 kb KpnI fragment shown to rescue *ftz* mutants (Hiromi, 1985). UPS, upstream element; N, neurogenic element, Z, zebra element. Transcription start site indicated by the black arrow below the zebra element. Black boxes, *ftz* exons. (B) Expanded view of the zebra element. Positions of gRNAs used indicated with purple triangles. To produce the arms of the HDR templates, regions flanking the zebra element were amplified using the primers indicated with red and blue arrows. The regions removed in full *ftzΔ*Z and partial *ftzΔ*Zp deletions are indicated by black lines below the diagram of the intact region. (C) Sequences of the final full and partial deletions. In the full deletion, the zebra sequences are replaced by an AvrII site (gold box). The pink box contains bases that constitute an indel in *ftzΔ*Zp. (D) Representative gel showing the PCR products of the region surrounding the zebra element produced from single fly preparations. Lanes: 1, DNA ladder; 2, control fly (*Dr/TM3*) with no deletion; 3, heterozygous fly from deletion line 1; 4, heterozygous fly from deletion line 2; 5, heterozygous fly from the partial deletion line; 6, homozygous fly from the partial deletion line; 7, heterozygous fly from the viable full deletion line (*Δ*Z); 8, homozygous fly from the viable full deletion line; 9, heterozygous fly from deletion line 5; 10, homozygous fly from deletion line 5; 11, heterozygous fly from deletion line 6.

One of the first genomic regions controlling early embryonic gene expression to be identified using the transgenic reporter gene approach was that of the pair-rule gene *fushi tarazu* (*ftz*) (Hiromi *et al.* 1985; Hiromi and Gehring 1987). *ftz* is expressed in a seven-stripe pattern in *Drosophila* embryos in the primordia of the alternate parasegments missing in *ftz* mutants (Hafen *et al.* 1984). The importance of precise control of *ftz* expression in stripes was highlighted by the finding that mis-expression of *ftz* throughout the embryo results in lethality (Struhl 1985). In work that was groundbreaking at the time, Hiromi *et al.* (1985) identified three major CREs within a 10 kb genomic fragment that was sufficient to rescue *ftz* mutants (see Figure 1A). The promoter proximal zebra element (Z) directed expression in seven *ftz*-like stripes, whereas the neurogenic element (N) directed expression in specific cells in the developing central nervous system (Hiromi *et al.* 1985; Doe *et al.* 1988). At the distal end of the 10 kb rescue fragment, Hiromi *et al.* identified an upstream element (UPS) that enhanced seven-stripe expression and which mediates autoregulation by Ftz and its partner Ftz-F1 (Hiromi and Gehring 1987; Pick *et al.* 1990; Yu *et al.* 1997). Further analysis of the zebra element identified short regions that activate or repress striped expression (Dearolf *et al.* 1989b; Topol *et al.* 1991), as well as a portion of the zebra element that directs broad expression in the domain corresponding to *ftz* stripes 4–7 via direct binding of Caudal (Cad) (Dearolf *et al.* 1989a), which had previously been shown to be required for posterior *ftz* stripe expression (Macdonald and Struhl 1986). These early studies led to the model that *ftz* expression is activated in all seven stripes via the zebra element, with interstripe repression playing a major role in stripe formation (Edgar *et al.* 1986; Ingham and Gergen, 1988; Carroll 1990). Following this, striped expression is maintained by autoregulation via the UPS (Hiromi and Gehring 1987; Pick *et al.* 1990).

Later studies questioned this simple model. For example, the demonstration that *hairy* and *ftz* stripes do not arise in complementary spatiotemporal patterns, along with a lack of biochemical evidence that Hairy binds to the zebra element as a *trans*-acting factor, ruled out Hairy—the best candidate for interstripe repression—as playing a role in stripe establishment; rather, Hairy as well as Even-skipped (Eve) appear to maintain the *ftz* pattern once it is established (Yu and Pick 1995). Identification of CREs that direct expression of single *ftz* stripes, similar to the classic *eve* stripe-specific elements (Small *et al.*^1991^, ^1992^, ^1996^; Arnosti *et al.* 1996) raised further doubts about seven-stripe elements establishing *ftz* stripes (Calhoun and Levine 2003; Schroeder *et al.* 2004, 2011), as did detailed analysis of pair-rule stripes in wild type and mutant embryos (Clark and Akam 2016). Rather, they supported the model that *ftz* expression is initiated in individual stripes by maternal or gap proteins interacting with stripe-specific CREs, followed by refinement and maintenance of stripes by pair-rule proteins interacting with seven-stripe CREs (Yu and Pick 1995). Stripe-specific elements were identified for *ftz* stripes 1 + 5, 2 + 7, and 3 + 6/7 but not for stripe 4 (Schroeder *et al.* 2011). Schroeder *et al.* (2011) concluded that there is no stripe 4 CRE and proposed that the zebra element was the only CRE responsible for directing the initial expression of this *ftz* stripe. However, sequential deletions of the zebra element in reporter transgenes failed to identify any region of the zebra element directing expression in only stripe 4, as loss of expression in stripe 4 coincided with the loss of expression in the other stripes (Dearolf *et al.* 1989a, 1989b).

Here, we evaluated conclusions drawn about *ftz* regulation from the use of reporter gene analysis in transgenic organisms, by precisely deleting the zebra element from its endogenous locus using CRISPR-Cas9 genome editing. Contrary to expectations that this CRE is either the major driver of expression of all seven *ftz* stripes (Hiromi *et al.* 1985) or that it is the sole driver of *ftz* stripe 4 initiation (Schroeder *et al.* 2011), we found that levels of expression of *ftz* stripe 4 were reduced but not fully lost in homozygous mutant embryos, whereas expression of other stripes was only marginally reduced. Animals homozygous for this genomic deletion were viable and fertile, with frequent defects observed in regions of larvae and adults corresponding to the stripe 4 expression domain. The *ftzΔZ* homozygous line will be useful for further studies of Ftz function, as target gene expression was altered specifically in this portion of the embryo, providing an internally controlled environment—within a single embryo—to monitor gene expression. Overall, our results show that even for a well-studied gene like *ftz*, our understanding of the qualitative and quantitative contributions of CREs to endogenous gene expression is far from complete.

## Materials and Methods

### Transgenic plasmids

Genomic fragments for reporter constructs were amplified with NEB Q5 DNA polymerase (catalog number: M0491S) following product specifications; annealing temperatures were predicted with NEB Tm Calculator (https://tmcalculator.neb.com/#!/main). See Supplementary Table S1 for the sequences of all primers and probes used in this work. *Zebra-73* was amplified with MF118 and MF119 and contains sequence from -670 to -74. *Zebra-40* was amplified with MF118 and MF116 and contains sequences from -670 to -41. MF116, MF118, and MF119 all contain XbaI adapter sites on the 5′ terminus. PCR products were inserted into the XbaI site of vector *placZ-attB* using Pyrite cloning (Fischer *et al.* 2018). Plasmids were sequenced with SeqF and SeqR primers and sent to Rainbow Transgenic for embryonic microinjection into Bloomington Stock Center line 9740, which contains a second chromosome phiC31 docking site at Chromosome 2, 57F5, 2R: 21645971.

### Guide RNA and homology directed repair template

Genomic targets for guide RNAs (gRNAs) matching the *ftz* zebra element were identified using CHOPCHOP (Labun *et al.* 2019) and the CRISPR Efficiency Predictor (Housden *et al.* 2015; Perrimon 2020). DNA from *nos-Cas9* flies was sequenced to check for polymorphisms in the zebra element and surrounding regions to ensure a perfect match between gRNAs and the genome of flies in which targeting was to be carried out (Supplementary Figure S1). Two gRNAs were selected and inserted into *pCFD4-U6:1_U6:3tandemgRNAs* (Addgene no. 49411; Port *et al*. 2014), one matching *ftz* -634 to -614, the other matching -211 to -191. The Homology Directed Repair (HDR) template was generated by two PCR reactions using *nos-Cas9* genomic DNA and NEB Phusion HF DNA polymerase (catalogue number MD530S) to amplify the left and right homology arms. One reaction amplified sequence upstream of the zebra element (-2296 to -670), adding an AvrII site at the 3′ end to produce the left homology arm. The second reaction amplified sequence from −73 to +1755, adding an AvrII site to the 5′ end to produce the right homology arm. These fragments were annealed, amplified by PCR, inserted into Promegea pGEM-T easy (catalogue number A1360) and verified by sequencing. The plasmid was digested with AvrII and reannealed to generate a repair template containing 3455 bp of the *ftz* region 1627 bp upstream of the zebra element and 1828 downstream of the zebra element, with the zebra element (-671 to -74) replaced by a single AvrII site. Embryos were microinjected by Rainbow Transgenic Flies with an injection mix containing *pCFD4-U6:1_U6:3tandemgRNAs* (100 ng/µl), *pCFD3-ebony* (100 ng/µl), and *pGEM HDR* (250 ng/µl) vectors. The vector *pCFD3-ebony* (Addgene no. 83380) contains an *ebony* guide for Co-CRISPR (Kane *et al.* 2017).

### Genetics

Fly stocks used were: y[1] M{w[+mC]=nos-Cas9.P}ZH-2A w[*] (BDSC 54591), referred to as *nos-Cas9*; and P{ry[+t7.2]=ftz-lacZ.ry[+]}TM3, e Sb[1] ry[*]/Dr[Mio] (BDSC 3218), referred to as *Dr/TM3, Sb*. *w^1118^* was used as wild type. Flies were reared at room temperature or at 25°C. To generate deletion lines, each injected *nos-Cas9* adult was crossed to 3 *Dr/TM3, Sb* flies. If the G0 cross produced ebony progeny, 10 F1 *Sb* individuals from that cross were used to setup F1 crosses (1 F1 *TM3, Sb* × 3 *Dr/TM3, Sb*). For G0 crosses producing no ebony progeny, six F1 crosses were set up. After individuals from the F1 crosses mated and laid eggs, each F1 *TM3, Sb* parent was screened for mutations. F2 *TM3, Sb* progeny from F1 individuals that had a deletion were crossed to each other to generate balanced lines. Two additional backcrosses were carried out to eliminate the *nos-Cas9* X chromosome.

To identify genomic deletions, gDNA was prepared from single flies (Gloor and Engels 1992). One microliter of the single fly preparation was used in a PCR reaction with one of the following primer sets, each of which flank the zebra element: zebra1 (positions −754 to −733) and zebra2 (positions +710 to +731), or Dm zebra fullL (positions −1542 to −1520) and Dm zebra fullR (+1121 to +1142). The wild type and the deletion PCR products were expected to be 1485 and 883 bp for the zebra1,2 primer pair (data not shown) and 2685 and 2089 bp with the zebraFullL, R primer pair. PCR bands indicating deletions were sequenced. The co-CRISPR strategy was effective in identifying HDR events: of 23 fertile G0 adults recovered, 9 (75%) that produced ebony offspring also produced *ftz* deletions as opposed to 5 (45%) for those not producing ebony progeny. For 10 of the 14 (71%) G0 that had an HDR event, at least half of the progeny carried the deletion.

### Gene expression analysis

For colorimetric *in situ* hybridization, digoxigenin-labeled probes were used following standard protocols (Kosman *et al.* 2004) and imaged using a Zeiss Microscope Axio Imager M1 microscope. For fluorescent *in situ* hybridization chain reaction (HCR), embryos were simultaneously stained for *ftz* and *eve* mRNA using a two-stage HCR protocol previously optimized for *Drosophila* (Choi *et al.* 2014; Surkova *et al.* 2019) except that the denaturing step with proteinase K was replaced by heating embryos barely submerged in phosphate-buffered saline, 0.1% Tween (PBST) in a microcentrifuge tube to 90°C for 5 minutes. Regions of *ftz* and *eve* transcripts that do not match other transcripts in the *D. melanogaster* transcriptome (BLAST, FlyBase Dmel Release 6.26), were submitted to Molecular Instruments for probe design. Hairpin amplifier pairs were: *ftz*, Alexa Fluor 488; *eve*, Alexa Fluor 546. All HCR buffers were made in the lab using previously published specifications and storage conditions (Choi *et al.* 2014). Nuclei were stained with 5 ng/µl (1:1000 in PBST) Hoechst 34580 (ThermoFisher) for 8–10 minutes, rinsed 3× with PBST, and washed 3× in PBST for 15 minutes. Embryos were then washed in PBS for 15 minutes before being mounted in Prolong gold antifade mountant (ThermoFisher). Visualization, background processing (Supplementary Figure S2), and quantification of HCR images was based on (Surkova *et al.* 2008b, 2008c, 2013b, 2019) and are described in the Supplementary Materials and Methods in detail. In short, the fluorescent integration of each stripe was determined using the width of each stripe and the fluorescent intensity to calculate the area under each stripe in the central 10% of the embryo. Thus, both stripe width and stripe intensity are an intrinsic part of the calculation.

## Results

### Deletion of the *ftz* zebra element

To test how the zebra element contributes to endogenous *ftz* gene expression, we used CRISPR-Cas9 to generate a precise deletion of this genomic region. A common strategy is to replace deleted regions with a reporter gene expressing a visible marker, such as *3XP3-GFP* (Berghammer *et al.* 1999), which greatly facilitates identification of genome editing events. However, we reasoned that insertion of a strong enhancer and promoter into the zebra-element region could impact expression of *ftz* itself and thereby confound effects of the zebra element deletion. Thus, although our strategy required screening individual flies by PCR, we designed an HDR template that removes the entire zebra element, replacing it only with a single AvrII site (Figure 1B).

The HDR template leaves intact the *ftz* basal promoter, including a GAGA site downstream of the deletion, in order to avoid impacts on basal promoter activity. The neurogenic element is left intact upstream of the deletion (Figure 1B). The mutation generated in this way removes 597 bp (-670 to -74, Figure 1B). G0 individuals were crossed to *Dr/TM3, Sb* flies, and F1 individuals were screened for the presence of a deletion, identifying 75 F1 flies carrying a deletion detectable by PCR. From these, we generated six balanced lines (see *Materials and Methods*). Genotyping demonstrated that five lines carried the exact zebra element deletion described above (*ftzΔZ*, Figure 1C). One line carried a partial deletion (*ftzΔZp*) of 432 bp removed from the 5′ portion of the zebra element (-637 to -207). This deletion appears to have resulted from a nonhomologous end joining event following cleavage by both of the gRNAs, as it removed most of the region between those cleavage sites as well as 3 bp 5′ to the first gRNA (Figure 1, B and C). The 3′ end of the deletion occurred within the second gRNA and removes 4 bp from the 5′ end of the gRNA cleavage site.

Although we expected that deletion of the zebra element would result in homozygous lethality, we recovered homozygous viable adults for two lines with the complete deletion and one with the partial deletion. One of the full deletion lines was homozygous sterile, but the other was fertile. As these mutations were balanced over a *TM3, Sb* chromosome, the homozygotes were identified by the absence of *Sb* and verified with PCR (Figure 1D; see *Materials and Methods*); only the wild type fragment (2685 bp) was amplified from control flies (balancer line, Figure 1D, lane 2). Amplification of genomic DNA from *Sb* adults expected to be heterozygous for *ftzΔZ* generated two fragments: the wild type and the expected smaller fragment (2089 bp; five independent *ftzΔZ* lines, Figure 1D, lanes 3, 4, 7, 9, and 11). Amplification of genomic DNA from heterozygotes for *ftzΔZp* generated the wild type fragment and an additional fragment that was slightly larger than that seen for *ftzΔZ* (2252 bp, Figure 1D, lane 5). DNA isolated from flies identified as homozygotes by virtue of absence of the *Sb* marker produced only the smaller fragments (*ftzΔZ* lines, Figure 1D, lanes 8 and 10; *ftzΔZp*, Figure 1D, lane 6). Sanger sequencing was repeated on individual adult flies lacking the *Sb* marker, verifying homozygosity for the deletion (Supplementary Figures S3 and S4). Complementation tests indicated that lethality and sterility seen in four of the six full deletion lines resulted from off-target effects (Supplementary Table S2), and we proceeded to characterize the homozygous viable and fertile *ftzΔZ* and *ftzΔZp* lines. Taken together, these results demonstrate that the *ftz* zebra element is not necessary for *Drosophila* viability or fertility.

### The *ftz* zebra element is required for development of a single body segment

Since deletion of the *ftz* zebra element did not result in lethality, we were able to establish and maintain true-breeding lines of *ftzΔZ*^*−*^^*/*^^*−*^ and *ftzΔZp^−^^/^^−^* for phenotypic analysis. Many *ftzΔZ^−^^/^^−^* flies survive to adulthood, but viability is reduced compared with wild type controls (Supplementary Table S3). Although ∼60% of eggs laid by controls eclosed to adulthood, only ∼30% of *ftzΔZ^−^^/^^−^*eggs developed to adults, with losses at each stage — egg hatching, pupation, and eclosion - contributing to this decline. In contrast, *ftzΔZp* homozygotes displayed hatching, pupation and eclosion rates similar to controls.

**Figure 2:**
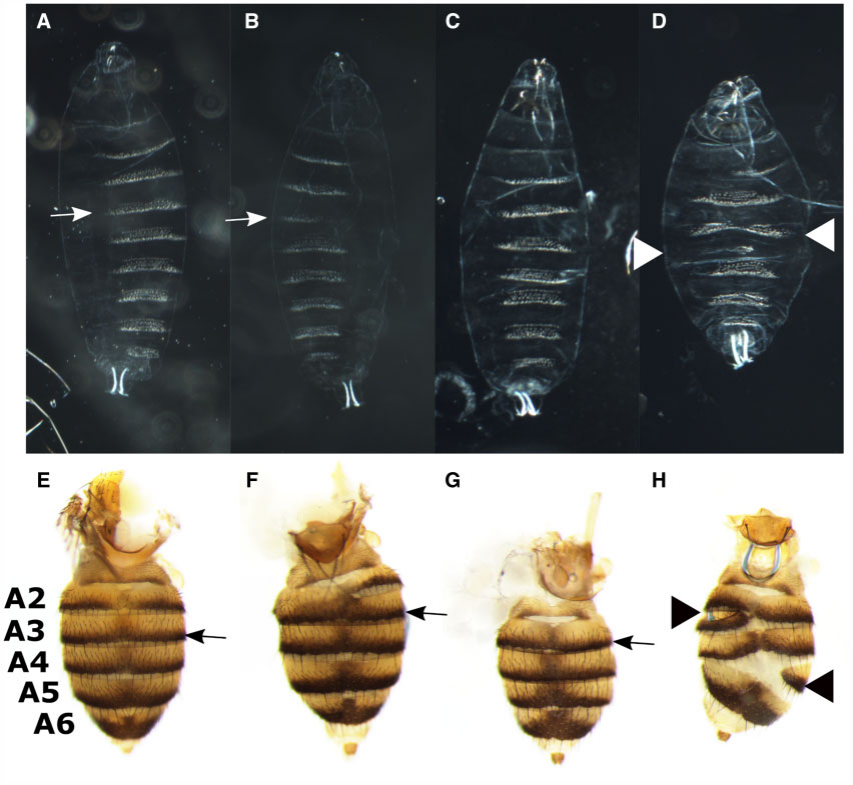
*ftzΔZ* larvae and adults have abdominal defects and are missing a single segment. Cuticles (A–D) or abdomens (E–H), anterior is up. (A–D) Larval cuticles from *ftzΔZp* (A–B), *ftzΔZ* (C–D). White arrows indicate denticle belt A3, and white arrowheads indicate abnormal denticle belts. *ftzΔZp* flies produce some cuticles that are wild type-like (A). Both *ftzΔZp* and *ftzΔZ* produce cuticles with the A3 denticle belt partially (B) or completely (C) missing. *ftzΔZ* also produces cuticles with defects in multiple denticle bands (D). Adult abdomens of *w^1118^* controls (E) or *ftzΔZ* homozygotes (F–H). Black arrows indicate the darkly pigmented region of segment A3, and black arrowheads indicate abnormal segments. Adult flies from *ftzΔZp* and *ftzΔZ* exhibit a similar range of phenotypes: wild type-like (not shown), partially (F) or fully (G) missing portions of segments A2 and A3, or multiple segments affected (H).

*ftzΔZ* and *ftzΔZp* homozygous larvae displayed a range of phenotypes, most involving segment A3 (Figure 2 and Supplementary Table S4). The most common phenotype for *ftzΔZ^−^^/^^−^* larvae was the absence of part (Figure 2B) or all (Figure 2C) of the denticle belt on segment A3, with other segments indistinguishable from wild type controls (*n* = 117: 84% missing all of the A3 denticle belt, 1% missing part). Some larvae (15%) displayed defects in additional denticle belts (Figure 2D and Supplementary Table S4), most commonly partial or complete loss of A5 denticles (11%), and a few (3%) showing defects in multiple denticle belts or in just A6 or A8 (1%). No normal cuticles were observed for *ftzΔZ* homozygous larvae. For *ftzΔZp* homozygotes, an almost equal mix of partial and full loss of denticle belt A3 was observed (*n* = 52: 44% each). None displayed defects in multiple segments and some cuticle preparations had wild type-like appearance (12%).

Similarly, adults homozygous for *ftzΔZ* or *ftzΔZp* eclosed with a range of phenotypes impacting segments A2 and A3, as well as more posterior segments (Figure 2, E–H, Supplementary Figure S5 and Table S5). *ftzΔZ^−^^/^^−^* adult flies were uniformly smaller in size than wild type adults, with smaller abdomens than wild type controls. Abdomens of *ftzΔZ^−^^/^^−^* flies (*n* = 639) either partially (18%) or completely (60%) lacked either A2 or A3 (Figure 2, F and G; Supplementary Figure S4, B and C and Table S5). The segment remaining most often had an A3 sternite bristle pattern, but sometimes had an A2 pattern or an ambiguous appearance. The ambiguity of identity seen here is reminiscent of the ambiguity seen in *hopscotch* (*hop*) mutants, which also survive to adulthood, missing a single segmental region, although *hop* mutants impact segments A4/A5 (Perrimon and Mahowald 1986). Abdomens of other *ftzΔZ^−^^/^^−^* flies (20%) displayed defects in multiple segments, with the posterior segments most severely affected (Figure 2H, arrowheads). A small number of adult abdomens had wild type-like appearance (2%). *ftzΔZp^−^^/^^−^* adults (*n* = 834) showed a similar range of phenotypes: 39% were missing part and 9% were missing all of A2 or A3, 50% were wild type-like, and 2% had defects in segments in addition to or other than A2 and A3.

In sum, these experiments demonstrate that the *ftz* zebra element plays a major role in the development of segments A2 and A3 while also impacting regions posterior to them. However, it appears to be fully dispensable for the formation of the thoracic and A1 segments. *ftzΔZp^−^^/^^−^* behaves as a weaker allele than *ftzΔZ^−^^/^^−^*, but the types of defects were similar, consistent with distributed regulatory information within the zebra element (Dearolf *et al.* 1989a, 1989b). Preliminary experiments indicate that a separate deletion of the zebra element to the -40 position results in the same range of phenotypes as observed for *ftzΔZ^−^^/^^−^* (data not shown). Finally, it appears that the presence of segment A2/A3 is not required for fly viability or for reproduction. The observed fertility is consistent with the gonads originating in segment A4 (Karch *et al.* 1985), which was not severely impacted in most *ftzΔZ^−^^/^^−^* homozygotes.

### *ftz* stripe 4 expression is reduced in *ftzΔZ^−^^/^^−^* mutants

**Figure 3:**
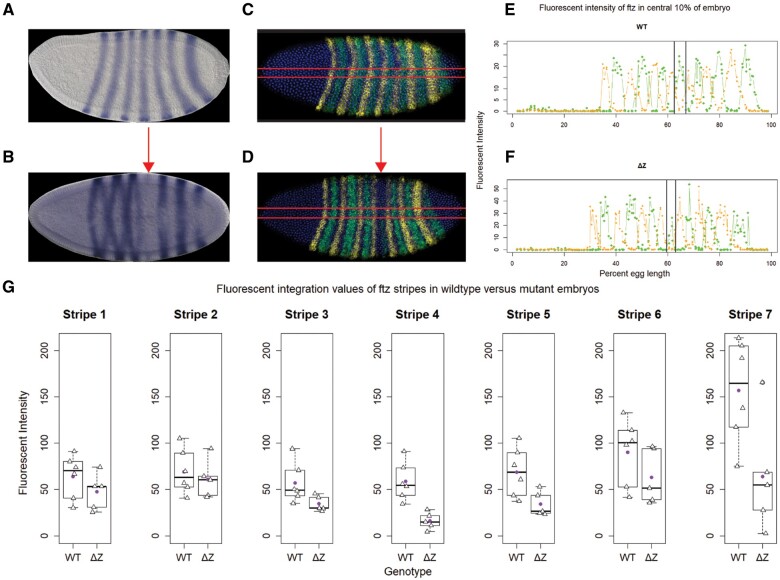
*ftz* stripe 4 is significantly reduced in *ftzΔZ^−/−^* mutant embryos. (A, B) Colorimetric *ftz* in situ hybridization. In wild type embryos (A), *ftz* is expressed in seven alternating stripes approximately four cells wide. Though all the stripes are present in *ftzΔZ* embryos (B), stripe 4 is weak (arrow). (C, D) Fluorescent in situ HCR (*ftz*, green; *eve*, yellow; nuclei, blue Hoechst). Qualitative differences (A, B) were also observed in HCR-stained embryos (C, wild type; D, *ftzΔZ*). Red lines indicate the approximate central 10% of the embryo’s dorsal-ventral axis from which centroid fluorescent signals are plotted in a 1D analysis along the anterior-posterior axis (E, wild type; F, *ftzΔZ*). Points within line graph represent centroids. Vertical lines indicate the boundaries of *ftz* stripe 4. (G) Fluorescent integration for each stripe in each embryo. Analysis of each stripe is indicated in a separate box plot. Stripe 4 is significantly reduced to 27.5% ± 18.5% in *ftzΔZ* embryos relative to wild type embryos (G; ± represents standard deviation; *P*-value = 0.0176; *n* = 6 wild type, 5 *ftzΔZ*). Triangles represent mean stripe 4 integrations from each embryo. Purple circle represents mean of means.

Although the zebra element was previously shown to direct expression of all seven *ftz* stripes in reporter-transgenes, the *ftzΔZ^−^^/^^−^* phenotype suggested that stripes were differentially affected in this mutant. To assess this, we analyzed *ftz* expression by *in situ* hybridization using digoxigenin-labeled probes. At the cellular blastoderm stage when *ftz* stripes peak, the expression of *ftz* stripe 4 was greatly decreased in *ftzΔZ^−^^/^^−^* embryos (Figure 3B, arrow). We next used fluorescent *in situ* HCR to determine the extent to which stripe 4 expression in *ftzΔZ^−^^/^^−^* mutants differed from wild type. Expression of *eve* was used as an internal control in these reactions and appeared unaffected by the deletion, as expected (Figure 3, C and D, yellow). In contrast, the change in *ftz* stripe 4 was evident by the gap in expression in the central region of the embryo (Figure 3D, arrow). Calculating the area under each stripe’s fluorescent signal intensity (see Supplementary Materials and Methods) revealed that stripe 4 was reduced to 27.5%  ± 18.5% of wild type levels in *ftzΔ^−^^/^^−^* mutants (*n* = 6 wild type, 5 *ftzΔZ; P*-value = 0.0176; Figure 3, E–G and Table 1). *ftz* stripe 4 is expressed in the primordia of segments A2 and A3, the segments most often affected in *ftzΔZ^−^^/^^−^* mutants (Figure 2 and Supplementary Tables S4 and S5). Comparing average stripe widths between wild type and *ftzΔ^−^^/^^−^* mutants also revealed that only stripe 4 was significantly reduced to about 53% the width of wild type. Notably, there was no statistically significant difference detected for the average central position of each stripe when comparing wild type to *ftzΔ^−^^/^^−^* mutants. Further, the positioning of *ftz* stripes relative to *eve* was the same in the *ftzΔ^−^^/^^−^* mutants as compared with wild type (data not shown). However, it is possible that alterations in the timing of *ftz* expression may also affect the phenotypes produced. In sum, these results suggest that for stripe 4, ∼25% of wild type is very close to the lower threshold of *ftz* transcript that is both necessary and sufficient to direct segmental development.

**Table 1:** Quantitative analysis of levels of individual *ftz* stripes

Stripes	*w^1118^* Integrations	*ftzΔZ* Integrations	Percentage (*ftzΔZ*/*w^1118^*)	*P-*value	*P*-adj
Stripe 1	63.9 ± 23.6	47.4 ± 19.6	74.2 ± 41.1	0.238	0.609
Stripe 2	69.1 ± 24.2	60.9 ± 21.1	88.2 ± 43.4	0.564	0.609
Stripe 3	56.9 ± 21.6	34.6 ± 8.37	60.8 ± 27.4	0.0546	0.218
Stripe 4	58.6 ± 20.6	16.1 ± 9.22	27.5 ± 18.5	0.00251	0.0176
Stripe 5	68.9 ± 26.5	34.1 ± 13.4	49.6 ± 27.2	0.024	0.144
Stripe 6	90.3 ± 35.7	63.2 ± 29.8	70 ± 43.1	0.203	0.609
Stripe 7	157 ± 55.5	63.9 ± 62.3	40.7 ± 42.2	0.0317	0.159

Consistent with changes in *ftz* stripe 4, the expression of two genes known to be regulated by Ftz, *sloppy-paired1* (*slp1*) and *engrailed* (*en*), were perturbed in the *ftz* stripe 4 domain of *ftzΔZ^−^^/^^−^* mutants (Supplementary Figure S6). As predicted by the Gergen lab, Ftz is required to repress *slp1* expression (Prazak *et al.* 2010; Hang and Gergen 2017). We found loss of *slp1* interstripe repression in *ftzΔ^−^^/^^−^* mutants, specifically in the domain impacted by *ftz* stripe 4, between *slp1* stripes 8 and 9 (Supplementary Figure S6, B and C). For *en*, a well characterized direct target of Ftz, a range of expression was observed: in some embryos, *ftz* stripe 4 expression was sufficient to generate a wild type-like *en* pattern (Supplementary Figure S6E); in others, *en* stripe 8 was weak but detectable (Supplementary Figure S6F); and, finally, in some embryos, *en* stripe 8 was undetectable (Supplementary Figure S6G). Occasionally, embryos displayed additional abnormalities in *en* expression, such as missing *en* stripes 6 and 8 and weak expression of stripes 10 and 14 (Supplementary Figure S6H), or a slight shift in position of an *en* stripe (Supplementary Figure S5, F and H). This is similar to shifts of *en* stripes after partial rescue of an *eve* mutant with stripe-specific CREs driving *eve* expression, which also correlated with loss of specific segments (Fujioka *et al.* 2002). Thus, the range of defects observed morphologically reflects the range of defects seen for target gene expression. The true-breeding *ftzΔZ* mutant lines, in which patterning is disrupted differentially in different regions within single embryos, provide a tool to examine the role of *ftz* in regulating other target genes.

### The zebra element contributes marginally to expression of other stripes

Although the decrease in *ftz* stripe 4 explained the phenotypic effects on A2/A3 in *ftzΔZ*^*−*^^*/*^^*−*^ mutants, transgenic reporters containing the zebra element were expressed in seven stripes (Hiromi *et al.* 1985; Dearolf *et al.* 1989b). What role does the zebra element play for these other stripes? Although not evident in colorimetric *in situ* experiments, at peak expression, levels of all seven stripes were lower in the mutant compared with wild-type controls in HCR labeled embryos (Figure 3G and Table 1). Interestingly, stripes 4, 5, and 7 showed the greatest reduction. Stripe 5 was reduced to 49.6% ± 27.2% and stripe 7 was reduced to 40.7% ± 42.2%. Though neither of these reductions was statistically significant after correcting for multiple comparisons, stripe expression level variance likely explains the range of defects seen in posterior abdominal segments of some *ftzΔZ^−^^/^^−^* mutant animals. For example, if *ftz* is reduced below the critical value for determination in either or both stripes 5 and 7 in addition to stripe 4 for some of the mutant embryos, it could result in the aberrant segmentation defects that are sometimes observed in segments other than A2/A3. Since the majority of mutant embryos develop with only A2/A3 parasegment defects, we suggest that the average *ftz* expression for stripes 5 and 7 are above the threshold level required for segmental development in most *ftzΔZ^−^^/^^−^* mutants, though the precise value of the threshold required for segmentation in these stripes cannot be determined with this dataset. Posterior defects likely occur in the embryos expressing less than the critical value, although subtle alterations in timing of these posterior stripes may also impact morphology.

In summary, it appears that variation around the minimal level of *ftz* transcript required for segmentation causes the variation in phenotype. Given that the average level of expression in those stripes is higher than that of stripe 4, the number of embryos with expression low enough to cause an effect would be fewer, consistent with the observation that relatively few adults show defects in segments other than A2 or A3.

### The order of *ftz* stripe activation is perturbed in *ftzΔZ*^*−*^^*/*^^*−*^ mutants

**Figure 4:**
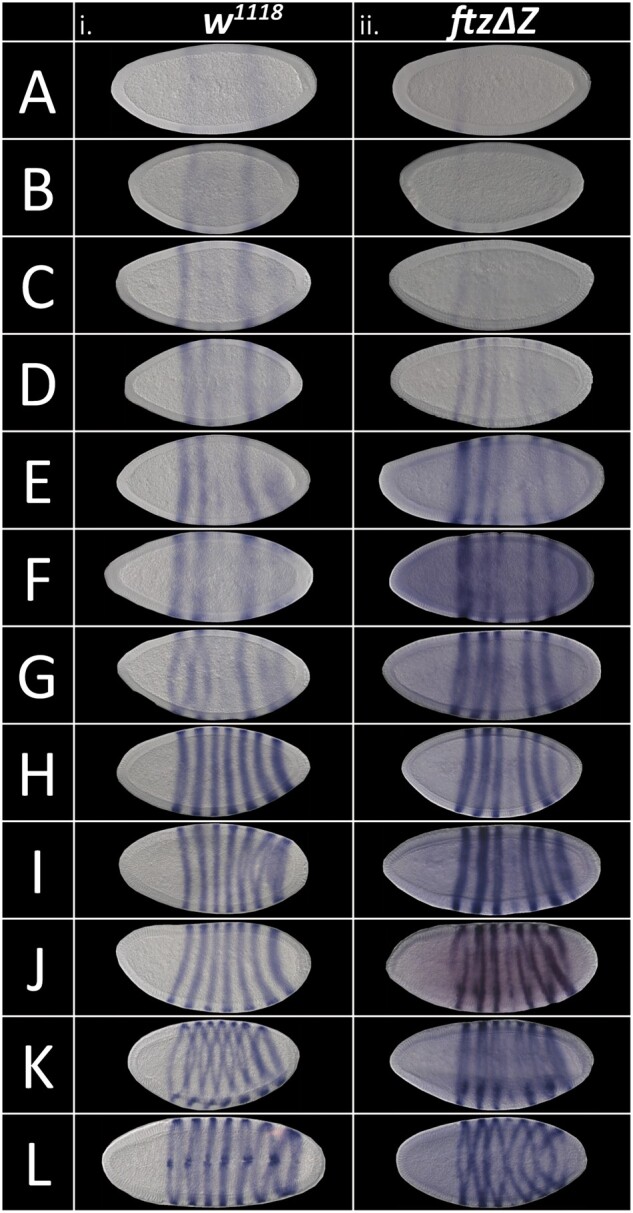
*ftz* stripes arise in different orders in *ftzΔZ* mutants than in wild type embryos. Time course of *ftz* stripe activation with colorimetric *in situ* hybridization in (i) wild type and (ii) *ftzΔZ* embryos. Embryos oriented anterior left, dorsal up with the following exceptions: L(i). and L(ii). are facing the ventral side. The relative *ftz* stripe intensity, degree of nuclear elongation, and degree of membrane deposition was examined to assess the developmental stage of each embryo so that embryos at similar stages could be compared. In wild type (i): A: stripes 1 and 5 start to appear. B: stripes 2 and 3 start to appear. C: A combined stripe 6/7 starts to appear without boundary separating 6 from 7. D: stripe 2 is solid and 6/7 is stronger than C. E: Stripes 6 and 7 start to resolve, and expression is stronger on the ventral side. F: Stripes 6 and 7 are distinct and surround the embryo. G: Stripe 4 starts to appear, completing the full 7 stripe pattern. Stripes 3 and 4 are very faint at this stage. H: Peak expression of all seven stripes. I and J: *ftz* signal may start to degrade. K: Invagination of the ventral and cephalic furrows. L: Slightly later stage embryo, transitioning to GBE. In *ftzΔZ* (ii): A: stripe 1 starts to appear; unlike wild type (i), stripe 5 does not start to appear. B: Stripes 2 and 3 start to appear. C: Stripes 5 and 6 appear very faintly. D: Existing stripes become a little stronger. Unlike wild type, stripe 7 does not appear at this stage. E: Stripes 1, 2, 3, and 6 develop more. F: Same pattern as E but stronger. G: Stripe 7 starts to appear. Unlike wild type, stripe 4 does not appear yet. H: Stripe 4 starts to appear. The other six stripes become more defined. I: Stripe 4 continues to develop. J: Peak of *ftz* signal. K: Invagination of the ventral and cephalic furrows. Unlike wild type (i), the zebra deletion mutant does not develop a strong stripe 4. L: Like wild type; ventral furrow about to transition to GBE.

The *ftz* seven-striped pattern develops via a dynamic, sequential addition of stripes (Yu and Pick 1995; Surkova *et al.* 2008a). We therefore considered the possibility that, in addition to impacting quantitative levels of stripe expression, the pattern of stripe development might be perturbed in *ftzΔZ^−^^/^^−^* mutants. Though wild type embryos show some variation in the order of *ftz* stripe activation (Surkova *et al.* 2008a), we found the generalized order to be as previously observed (Yu and Pick 1995): first, stripes 1 and 5 appear as the nuclei undergo elongation (Figure 4i-A). Stripes 2 and 3 appear next (Figure 4i-B), followed by a combined stripes 6 and 7 that appears initially on the ventral side of the embryo and progresses dorsally (Figure 4i-C). At this point, the nuclei are fully elongated and plasma membrane deposition begins at the apical pole, continuing towards the cortical cytoplasm. Stripes 1, 2, 3, and 5 become more established, whereas stripes 6 and 7 resolve into separate stripes (Figure 4i, E and F). Stripe 4 appears last, completing a pattern of all seven stripes (Figure 4i-G). Levels of expression in all seven stripes increase as the plasma membrane extends to the basal plane of the nuclei (Figure 4i-H). This pattern is maintained as membrane deposition completes cellularization (Figure 4i, I and J). When the ventral and cephalic furrows form, the embryo has entered the germband extension (GBE) phase, and *ftz* stripes begin to fade (Figure 4i, K and L). In summary, the *ftz* stripes form in the following order in a wild type background: 1 + 5, 2 + 3, a combined 6/7 which then resolves into separate stripes, and finally 4.

Initiation of *ftz* stripe formation in *ftzΔZ*^*−*^^*/*^^*−*^ mutants differed in several ways from the wild type pattern. First, the order in which stripes arose differed: stripes 1 and 3 appeared first (Figure 4ii, A and B), then stripe 2 (Figure 4ii-C) before posterior stripes 5 and 6 (Figure 4ii-D). Stripes 1, 2, 3, 5, and 6 continued to increase in intensity (Figure 4ii, E and F) and were joined by stripe 7 (Figure 4ii-G). Unlike wild type, in which stripes 6 and 7 arise as a fused stripe, stripe 7 appeared separately from stripe 6 in *ftzΔZ^−^^/^^−^* mutants. Stripe 4 appeared last, as in wild-type embryos, completing the set of seven stripes that increase in intensity until GBE begins (Figure 4ii, H–J). In summary, *ftz* stripes arose in the following order in *ftzΔZ^−^^/^^−^* mutants: 1, 3, 2, 5 + 6, and 7, with 4 being the last. For *ftzΔZp^−^^/^^−^*, the order was again slightly different: 1 + 5, 3 + 6, 2, and 7, with 4 being the last one (data not shown). The major differences in the appearance of *ftz* stripes are in the posterior region of the embryo, most notably stripes 6 and 7, which arise in a coordinated fashion in wild type embryos but independently and in a different temporal order in *ftzΔZ^−^^/^^−^* mutants.

### Refining the boundary between the zebra patterning element and basal promoter

**Figure 5:**
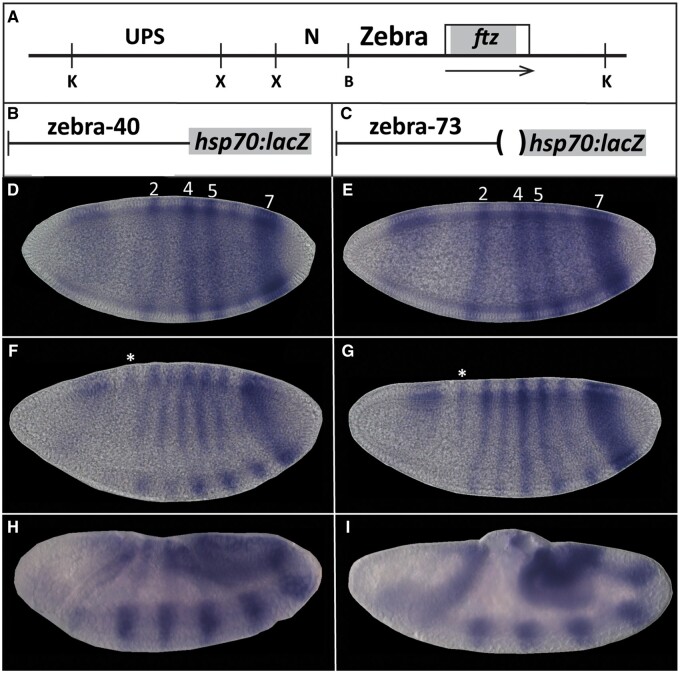
Zebra element transgenes striped expression pattern. (A) Schematic of *ftz* genomic locus with CREs identified by Hiromi (1985) shown; UPS, neurogenic element (N), and zebra element. Restriction enzymes originally used to map these canonical elements are abbreviated below the line; KpnI (K), XbaI (X), and BalI (B). (B, C) Schematic of zebra element transgenes: Transgenes differ at the 3′ end. *zebra-40* includes zebra element sequences up to 40 bp upstream of the *ftz* transcription start site; *zebra-73* includes zebra element sequences up to 73 bp upstream of the *ftz* transcription start site, omitting a GAGA site present in *zebra-40*. (D–I) Expression of *lacZ* reporter genes using colorimetric *in situ* hybridization. (D, E) mid blastoderm, *ftz* stripes labeled; (F, G) late blastoderm, asterisk above dorsal stripe 1; (H, I), germband extension. Embryos oriented anterior, left; dorsal, up. Embryos were staged according to degree of membrane deposition and morphology.

The *ftzΔZ* deletion described above removed the zebra element from position -670 at the 5′ end, as defined by Hiromi *et al.* (1985) and Dearolf *et al.* (1989a, 1989b), to position -74 at the 3′ end (see Figure 1). Although the *ftz* basal promoter had previously been defined as requiring sequences to -40 (Dearolf *et al.* 1989b), our genome edit left intact additional sequence that includes a potential binding site for the chromatin remodeling protein GAGA Factor (Topol *et al.* 1991), which could influence basal promoter accessibility (Judd *et al.* 2021; reviewed in Chetverina *et al.* 2021). To ensure that the choice of the -74 position for the zebra element deletion did not inadvertently leave intact patterning information for *ftz* stripes, we generated two reporter genes: *zebra-40* includes sequences between -670 and -41 (*zebra-40*), whereas *zebra-73* includes sequences between -670 and -74. Each fragment was fused upstream of a basal *hsp70* promoter in vector *placZ-attB* and integrated into the *Drosophila* genome at a site on chromosome 2 previously shown to allow embryonic expression (Figure 5, A–C). These two reporter transgenes were expressed in seven-stripe patterns indistinguishable from each other and from the pattern previously reported for *ftz* zebra element transgenes (Hiromi *et al.* 1985; Dearolf *et al.* 1989b). At the mid-blastoderm stage, expression was detected in stripes, with stripe 7 being the strongest, stripe 6 barely detectable, stripes 5 and 4 clearly formed, stripe 3 emerging, stripe 2 well resolved, and stripe 1 not yet detectable (Figure 5, D and E). As previously noted, ectopic expression in the head region is observed for these zebra element reporters (Hiromi *et al.* 1985). At the cellular blastoderm stage, stripe 1 remained barely detectable, appearing more strongly on the dorsal side (Figure 5, F and G, asterisk) while all other stripes had clearly resolved, with stripe 7 remaining the strongest. By early- to mid-GBE (Figure 5, H and I), stripes 2–7 were well developed, with expression stronger in the mesoderm than ectoderm, as previously reported (Hiromi *et al.* 1985). Stripe 1 remained faint throughout. Thus, the region between -73 and -40 does not contribute to the striped pattern directed by the zebra element.

## Discussion

Much has been learned about the regulation of transcription using reporter constructs expressed in transgenic animals. However, due to their very nature, these experiments do not account for chromatin states that may impact utilization of identified CREs in their endogenous genomic locations. They also rarely address interactions between multiple CREs. These reporter transgenes identify genomic regions *sufficient* to direct spatiotemporal expression patterns but do not provide information about whether or to what extent these genomic regions are *necessary* for endogenous gene expression. Further, the fact that even exceedingly low levels of reporter gene expression can be detected above background in these assays can make the CRE appear quantitatively more important than it is in the native context. This is exemplified by our studies here, using CRISPR-Cas9 to remove the *ftz* zebra element from its endogenous genomic location (Figure 1). Based on previous experiments with transgenes, as well as those reported here (Figure 5), we thought it likely that this CRE would be necessary for *ftz* function. However, we found that homozygotes developed into fertile adults, and we were able to establish stable *ftzΔZ^−^^/^^−^* stocks (Figure 1D). Genetic manipulations of other segmentation genes also produced viable adult flies missing specific segments (Perrimon and Mahowald 1986; Howard *et al.* 1988; Fujioka *et al.* 2002). Thus, flies can develop to and survive as adults missing whole body regions, although their fitness may be compromised. Despite this, because the zebra element deletion most strongly impacted segments A2/A3 and gonads develop from segment A4 (Karch *et al.* 1985), we were able to generate fertile, true-breeding lines for this particular mutant.

Since zebra element transgenes drive expression at some level in all seven stripes, deletion of this CRE was expected to reduce or eliminate expression to some extent in all seven stripes. The fact that zebra element transgenes are more strongly expressed in the posterior stripes suggested that a deletion might have a bigger effect on those stripes. In our analysis, we did find that expression was reduced to some extent in all stripes, with a slightly larger but nonsignificant effect on stripes 5 and 7 over stripes 1–3 and 6. Thus, the zebra element plays a role in modulating all of the stripes but the major impact of deleting it was a large decrease in levels of stripe 4 expression (Figures 3 and 4). The original descriptions of the zebra element-directed transgenes revealed a mesodermal bias for these transgenes, with stripes being broader and less well-refined than UPS-driven transgenes (Hiromi and Gehring 1987; Dearolf *et al.* 1989a, 1989b). The combination of the zebra and UPS generated a strong seven-stripe pattern with sharp stripes, more reminiscent of endogenous *ftz* expression, suggesting a synergistic interaction between these CREs. The level of expression of the zebra plus UPS transgene was also more than additive (see Yu and Pick 1995). Similar synergistic interactions between the other *ftz* CREs remain to be elucidated. In sum, while reporter studies were able to identify the zebra element as sufficient to drive expression in all seven stripes, they could not determine whether or to what extent this element was necessary for *ftz* expression; this serves as an example of the conflation of necessity and sufficiency. Although we demonstrate this point here for the *ftz* gene, the same situation undoubtedly applies to “enhancer bashing” studies done for a wide cadre of developmentally regulated genes in *Drosophila* and other organisms.

Previous research using reporter transgenes identified stripe-specific CREs that direct expression of individual *ftz* stripes (Calhoun and Levine 2003; Schroeder *et al.* 2011). Whether these stripe-specific elements are the primary drivers of *ftz* stripe establishment, play secondary roles in the maintenance of individual stripes, are evolutionary remnants, and/or are redundant with the 2 seven-stripe elements (zebra and UPS) remains to be determined. Our findings here support the notion that the stripe-specific elements play the predominant role in the establishment of the *ftz* stripe pattern, as suggested by Schroeder *et al*. (2011). These researchers identified stripe-specific elements directing the expression of each of the *ftz* stripes, except for stripe 4. The only region tested that directed expression in stripe 4 was a portion of the zebra element (*ftz*-1). This fragment also directed expression in more posterior stripes, predominantly 5 and 7, with stripe 6 less apparent. This is similar to the results of Dearolf *et al.* (1989a, 1989b) in that the stripes were modulated above a general broad expression in the posterior region. Thus, a strong impact on stripe 4 was expected upon deleting the zebra element, based on the work of Schroeder *et al.* (2011). However, contrary to their prediction that zebra contained the only primary CRE for stripe 4 establishment, the deletion of the zebra element demonstrates that the zebra element contributes quantitatively to stripe 4 expression but is not the sole element directing its expression—the zebra element lacks full regulatory information for stripe 4. Additional CRE(s) directing the establishment of *ftz* stripe 4 must reside elsewhere, either within the autoregulatory UPS or, more likely, in a genomic region not yet studied. Clark and Akam (2016) have proposed novel *trans*-regulatory interactions among pair-rule proteins involved in refining and positioning *ftz*, as well as *odd*, stripe 4; perhaps the yet-to-be identified *ftz* stripe 4 CRE contains binding sites for these pair-rule proteins (Clark and Akam 2016).

Interestingly, transgenes capable of rescuing *ftz* mutants do not accurately recapitulate the order of *ftz* stripe activation (Yu and Pick 1995), suggesting that although they provide enough information to produce the final seven-stripe *ftz* pattern, additional information is missing from these transgenes. Similarly, for the two deletion mutants that we generated, *ftzΔZ^−^^/^^−^* and *ftzΔZp^−^^/^^−^*, the order in which stripes arose differed, and neither matched wild type (Figure 4). Notably, though order was perturbed in both *ftzΔZ^−^^/^^−^* and *ftzΔZp^−^^/^^−^* lines, the phenotype was more severe in *ftzΔZ^−^^/^^−^* than *ftzΔZp^−^^/^^−^.* Together, these results suggest that development is more flexible than might be expected—the wild type order of stripe formation is not necessary for survival, since both *ftzΔZ^−^^/^^−^* mutants and *ftz* mutant flies rescued with the transgenes are still viable, despite neither recapitulating the wild type order of stripe establishment.

Analysis of *ftz* null mutants, which behave as standard recessives, suggests that a 50% reduction of expression in heterozygous flies is sufficient for normal development. The finding that zebra element deletions have region-specific impacts on target gene expression levels (Supplementary Figure S6) allowed us to dissect the quantitative requirement for gene expression more finely. The loss of the zebra element reduces expression levels in *ftz* stripe 4 to a point very near the minimum needed to produce segment A2/A3. This teetering around the threshold is evidenced by variability in phenotype: most embryos fail to produce enough *ftz* in stripe 4 to make segment A2/A3, whereas others produce just enough to make all or part of A2/A3, and a few develop fully (Supplementary Tables S4 and S5). This would suggest that expression at ∼25% of wild type is the minimum level required for *ftz* to direct the development of segments. The threshold for other stripes may not be the same, and indeed we did observe some variability in segments posterior to A3, suggesting that the level of expression in stripes 5–7 may also be coming close to a threshold. We hypothesize that the variation of phenotypic variability from these mutants is a result of variation of *ftz* expression in stripes 5–7. It is possible that embryos with reduced *ftz* expression in one or more of these posterior stripes fails to specify segmental fate — much like what we believe is consistently occurring in the segment derived from *ftz* stripe 4. However, it is worth noting that the work shown here cannot rule out the possibility that subtle changes in the timing of *ftz* stripe establishment in *ftzΔZ* mutants may contribute to the phenotypes observed, though no statistical difference was detected for stripe positioning from this data set. Future studies, assessing the quantitative contributions of other *ftz* CREs, will allow us to more accurately refine the features of *ftz* gene expression that are critical for promoting morphological segmentation. This may reveal general rules about how the analog information of transcription factor gene expression connects to the digital information of developmental fate.

A great deal of redundancy is thought to be built into the regulation of expression for developmentally regulated genes (Hong *et al.* 2008). For *ftz*, in addition to the 2 seven-stripe elements, there are multiple stripe-specific elements that direct expression of each of the *ftz* stripes, with two stripe-specific elements identified to date directing expression in stripe 7, although the exact spatial and temporal dynamics of each CRE may differ. Future work is required to compare the necessity, sufficiency, and potential shared and synergistic roles of these CREs, as well as to understand how these mechanisms changed during evolution as *ftz* was incorporated into the pair-rule gene network, having arisen as a *Hox* gene with a very different ancestral expression pattern (Heffer *et al.* 2013). To this end, the *ftzΔZ* mutants, and perhaps future *ftz* enhancer deletion mutants, could serve as genetic systems with “intra-embryo” internally controlled environments for studies of the role of *ftz* in regulating other target genes. Clearly, analysis of CREs using transgenes has provided much insight into this process. However, our studies, in keeping with those of others (Delker *et al.* 2019), highlight the importance of examining CRE function not only through transgenes, but also by functional analysis in the native context of the endogenous gene.

## Data availability

Fly lines and plasmids are available upon request. The authors affirm that all data necessary for confirming the conclusions of the article are present within the article, figures, and tables.

Supplementary material is available at *G3* online.

## Supplementary Material

jkab300_Supplementary_Data
