# Supplementary material for: The fushi tarazu zebra element is not required for Drosophila viability or fertility
Source: G3 (Bethesda). 2021 Aug 26;11(11):jkab300. doi: 10.1093/g3journal/jkab300 (PMC8527495; doi:10.1093/g3journal/jkab300)
Supplement: jkab300_Supplementary_Data [file jkab300_supplementary_data.zip › GENETICS-G3-2021-402710-s01.docx]

**Supplementary Materials and Methods**

**Confocal microscopy image acquisition, processing and data visualization**

All confocal images were taken on a Leica SP5X Laser Scanning Confocal microscope. The Leica Application Suite Advanced Fluorescence (LPA AF) software was used to control the hardware. To allow for quantitative analysis, all samples were imaged in the same session and with the same confocal microscope parameters for gain and offset. These calibrations were made following previously published specifications for quantitative purposes (Surkova et al. 2008a; Surkova et al. 2008b; Surkova et al. 2013c; Surkova et al. 2019). Briefly, the gain for *ftz-* and *eve*-detecting channels were set to be just below pixel saturation of the strongest observed sample stripe. Because the same gain and offset were used for all samples, the raw fluorescent intensity output could be extracted and used for comparative quantitative data analysis.

Three 1 µm stacking optical sections, averaged 8 times, were scanned through the lateral surface of embryos to a total depth of 2 µm using a 20x objective. Embryo positions were marked using the “mark and find” feature within the LPA AF software. Following fluorescent image acquisition, sagittal scans with a 63x objective were taken with white light to visualize the degree of nuclear elongation and membrane deposition in all marked embryos. This allowed for determination of which samples had completed or were near complete cellularization (Surkova et al. 2013b).

Fiji ImageJ software (Schindelin et al. 2012) was used to separate image stacks from LPA AF .lif files into discreet grayscale TIF files to be used as input for the ProStack software (Matveeva et al. 2006). Briefly, a previously published pipeline for ProStack allows for automated image processing designed for the quantification of fluorescent *Drosophila* embryo images (Surkova et al. 2008a). This automated pipeline detects the shape of the embryo and location of stained nuclei within it, rotates the embryo to be anterior left/dorsal up, crops the image, and detects fluorescent signals from each nuclear position. These nuclear positions are referred to as centroids, and each one is assigned an X and Y coordinate. The fluorescent signal intensity from each channel of each centroid is organized as a column delineated output file. This file can be opened in Excel or by other means, such as R, for further processing.

All data processing for visualization and statistical analyses of the ProStack output file were conducted in Rstudio (Team 2019). Fluorescent data from centroids in the central 10% (45%-55%) of the dorsal-ventral axis were used for one-dimensional (1D) analysis along the anterior-posterior (A-P) axis as previously described (Surkova et al. 2008a; Surkova et al. 2013c; Surkova et al. 2013b; Surkova et al. 2019). This allowed each centroid to be characterized by its X coordinate along the A-P axis, described as percent body length. These are the datapoints used in the HCR line plots shown here, and stripe domains can be clearly visualized with this 1D analysis.

It was previously described that the background fluorescent noise along this 1D analysis takes on a parabolic shape along the A-P axis (Surkova et al. 2013a). Background was removed from each channel and from each embryo with the following process: centroid data points below the stripe domains were used to create a quadratic model that was fit to the background noise. Each centroid data point was subtracted by the corresponding predicted background point from the model, resulting in the removal of the parabolic background noise. Datapoints that became slightly negative due to being less than the model were converted to values of zero.

Stripe domain boundaries of *ftz* were determined by first identifying the minimal fluorescent signal (threshold) of the stripe centroids. A script was written which analyzes each centroid from 0% to 100% body length; the left boundary of each domain was determined as the first centroid between a data point below the threshold and a data point above the threshold. Conversely, the right boundary of each domain was determined as the first centroid between a data point above the threshold and a data point below the threshold. This automated processing was manually curated to accurately determine the boundaries of each stripe in the 1D analysis.

The intensity of each *ftz* stripe was determined by integrating between each of the identified boundaries. This was calculated with the Area Under the Curve (AUC) function with the ‘trapezoid’ method in the ‘DescTools’ package in R (Signorell and al. 2020). Because this calculation accounts for both the amplitude and width of the stripe, it portrays the intensity from this 1D analysis, generating the value to be used for the quantitative stripe intensity comparisons between embryos of different genotypes.

Due to the highly dynamic nature of pair-rule gene expression during the cleavage cycle 14A interphase, embryos that were nearing complete cellularization and thus near peak expression were used to quantify *ftz* stripe domain expression levels. These embryos were selected from the confocal images on the basis of nuclear elongation, plasma membrane deposition, and relative max values of *eve* stripes. Selecting embryos in the same stage of cycle14A in this manner helped reduce how this stage’s dynamism could confound analysis. Previously published *eve* stripe dynamics convey a pattern of relative stripe intensity that correlates with the embryo’s developmental timepoint within cycle 14A (Surkova et al. 2019). By measuring the maximal *eve* intensity values for each stripe and determining the degree of membrane depositions, embryos were temporally categorized.

These criteria were used to select six wildtype embryos and five *ftzΔZ* embryos that were suitable for the quantitative dataset. The means of stripe intensity integration values from the wildtype and *ftzΔZ* mutant datasets were tested for statistical significance with the Student t-test. P-values were adjusted with the Holm’s method to correct for multiple comparisons. Normality of data was assessed statistically with the Shapiro-Wilk test.

**References cited in supplemental methods**

Matveeva A, Kozlov K, Samsonova M. 2006. Extraction of Quantitative Gene Expression Data from the Images of Gene Expression Patterns with ProStack and iSIMBioS. in *Proc of the 4rd TICSP Workshop on Computational Systems Biology (WCSB 2006)*, pp. 65–68, Tampere, Finland.

Schindelin J, Arganda-Carreras I, Frise E, Kaynig V, Longair M, Pietzsch T, Preibisch S, Rueden C, Saalfeld S, Schmid B et al. 2012. Fiji: an open-source platform for biological-image analysis. *Nat Methods* **9**: 676-682.

Signorell A, al. em. 2020. DescTools: Tools for descriptive statistics. R package version 0.99.32.

Surkova S, Golubkova E, Manu, Panok L, Mamon L, Reinitz J, Samsonova M. 2013a. Quantitative dynamics and increased variability of segmentation gene expression in the Drosophila Kruppel and knirps mutants. *Dev Biol* **376**: 99-112.

Surkova S, Myasnikova E, Janssens H, Kozlov KN, Samsonova AA, Reinitz J, Samsonova M. 2008a. Pipeline for acquisition of quantitative data on segmentation gene expression from confocal images. *Fly (Austin)* **2**: 58-66.

Surkova S, Myasnikova E, Kozlov KN, Pisarev A, Reinitz J, Samsonova M. 2013b. Preparation of Drosophila embryos for quantitative imaging of gene expression. *Cold Spring Harb Protoc* **2013**: 533-536.

-. 2013c. Quantitative imaging of gene expression in Drosophila embryos. *Cold Spring Harb Protoc* **2013**: 488-497.

Surkova S, Sokolkova A, Kozlov K, Nuzhdin SV, Samsonova M. 2019. Quantitative analysis reveals genotype- and domain- specific differences between mRNA and protein expression of segmentation genes in Drosophila. *Dev Biol* **448**: 48-58.

Surkova SY, Myasnikova EM, Kozlov KN, Samsonova AA, Reinitz J, Samsonova MG. 2008b. Methods for Acquisition of Quantitative Data from Confocal Images of Gene Expression in situ. *Cell tissue biol* **2**: 200-215.

Team RC. 2019. R: A language and environment for statistical computing. R Foundation for Statistical Computing.
