# Supplementary material for: The fushi tarazu zebra element is not required for Drosophila viability or fertility
Source: G3 (Bethesda). 2021 Aug 26;11(11):jkab300. doi: 10.1093/g3journal/jkab300 (PMC8527495; doi:10.1093/g3journal/jkab300)
Supplement: jkab300_Supplementary_Data [file jkab300_supplementary_data.zip › GENETICS-G3-2021-402710-s02.docx]

**Supplemental Table 1. Primers and probes used in this manuscript.**

| Name | Purpose | Sequence | Location |
| --- | --- | --- | --- |
| Dm ftz zebra gRNA1 | gRNA | TCAGGAATACGCGTGGCCGA | -634 to -614 |
| Dm ftz zebra gRNA2 | gRNA | CGCAGCAGGTAGGCACCGTA | -211 to -191 |
| zebra1 | PCR screen | ATTTTGGAAGTGCGTTTGTTG | -754 to -733 |
| zebra2 | PCR screen | TGACAGCTGACGAGGATTTCT | +710 to +731 |
| Dm Zebra fullL | PCR screen | TCCAAAACCGACGCATGAAGT | +1121 to +1142 |
| Dm Zebra fullR | PCR screen | CCAGAGTATGCGGATGGGGTA | -1542 to -1520 |
| Dm Zebra fullR2 | HDR construct | ATCGATCGCTGAGAACCCATC | +1734 to +1755 |
| Dm Zebra fullL2 | HDR construct | GAGCTCCGGATTCGCATTCTA | -2296 to -2275 |
| Dm_zebra homolRS | HDR construct | CACACACTCTCT CCTAGG CCAAGCAAGTGCGCAACTCCC | -690 to - 670 |
| zebra RS homol3 | HDR construct | AGAGAGTGTGTG CCTAGG GCTATAACGGCGAGCGTGTGC | -73 to -52 |
| *ftz* probe 3’ UTR | colorimetric *in situ* hybridization | GCCCTCTACTGTGCAACTCATC | +1584 to + 1606 |
| MF118 | F primer zebra-40 and zebra-73 | GCTCTAGACCACGAGGGCAAACAAAAAGCG | -670 to -649 |
| MF116 | R primer zebra-40 | GCTCTAGAAGAGAGCCCTCGGCACAC | -58 to -41 |
| MF119 | R primer zebra-73 | GCTCTAGAGCATAAATATCCCTGCGCATGACAC | -98 to -74 |
| SeqF | Sequencing pLacZattB inserts | AAATGCTTGGATTTCACTGG | N/A |
| SeqR | Sequencing pLacZattB inserts | GTTTGAATTGAATTGTCGCTCC | N/A |
| Zebra-40 transgene | Sequenced insert | TCTAGACCACGAGGGCAAACAAAAAGCGCAAACACGCGACCCTCGGCCACGCGTATTCCTGATCCCAGGGATCGGACGTAATGTTATCCTTTGGCCGCCCAGTGCCACGAAATAAATTCGGAGGGAAAGGGCATCGGGTTCCGGGAACAACTGGCAGCCAGTCTTCGGTGTTTTGCGCGCTGGCAAAAATCCAGAGAAATTTTTAGGGAACCATAAACGGGCCGGGGAAAAAGCCTCTGCGCCGAAGGAACGTTTTCAGCAACAGTTTACAGTTTTTATGTCTTTATGATTATTGCAATTAGAGGGAGATCGGCTGAGAGTCGCGCCCTCTCGCTCTGCGCACCTCATAGGTAGGCACCTCATGGCCGTAATTACTGCAGCACCGTCTCAAGGTCGCCGAGTAGGAGAAGCGCGCGGGCGGATAAATCGCGATGATAATGGGCGCGATGGGTAGGTAATAAGCCGCGCAGCAGGTAGGCACCGTACGGATAAAGTTGCCAGGACCTCGGATAACTTCCCCTCTCCGTGCCTGCAAGGACATTTCGCCGGAGGGGTGGCTGCGAACAGCAGGCGGCAAAGTGTCATGCGCAGGGATATTTATGCGCTATAACGGCGAGCGTGTGCCGAGGGCTCTCTTCTAGA |  |
| Zebra-73 transgene | Sequenced insert | TCTAGACCACGAGGGCAAACAAAAAGCGCAAACACGCGACCCTCGGCCACGCGTATTCCTGATCCCAGGGATCGGACGTAATGTTATCCTTTGGCCGCCCAGTGCCACGAAATAAATTCGGAGGGAAAGGGCATCGGGTTCCGGGAACAACTGGCAGCCAGTCTTCGGTGTTTTGCGCGCTGGCAAAAATCCAGAGAAATTTTTAGGGAACCATAAACGGGCCGGGGAAAAAGCCTCTGCGCCGAAGGAACGTTTTCAGCAACAGTTTACAGTTTTTATGTCTTTATGATTATTGCAATTAGAGGGAGATCGGCTGAGAGTCGCGCCCTCTCGCTCTGCGCACCTCATAGGTAGGCACCTCATGGCCGTAATTACTGCAGCACCGTCTCAAGGTCGCCGAGTAGGAGAAGCGCGCGGGCGGATAAATCGCGATGATAATGGGCGCGATGGGTAGGTAATAAGCCGCGCAGCAGGTAGGCACCGTACGGATAAAGTTGCCAGGACCTCGGATAACTTCCCCTCTCCGTGCCTGCAAGGACATTTCGCCGGAGGGGTGGCTGCGAACAGCAGGCGGCAAAGTGTCATGCGCAGGGATATTTATGCTCTAGA |  |
| Dm *ftz* CDS submitted to Molecular Instruments | HCR *in situ* | ATTCGCAAACTCACCAGCGTTGCGTGCACATCGCAGAGTTAGAGAAGAAATCTAGCAATACACATCCGATATGGCCACCACAAACAGCCAGAGCCACTACAGCTACGCCGACAACATGAACATGTACAACATGTATCACCCCCACAGCCTGCCGCCCACCTACTACGATAATTCAGGCAGCAATGCCTACTATCAGAACACCTCCAATTATCAGGGCTACTATCCCCAGGAGAGTTACTCGGAGAGCTGCTACTACTACAACAATCAGGAGCAGGTGACCACCCAGACTGTACCGCCCGTGCAACCCACCACCCCGCCGCCCAAGGCCACCAAGCGCAAGGCCGAAGATGATGCTGCTTCCATCATCGCCGCCGTGGAGGAGCGACCCAGCACACTGAGGGCTCTGCTCACCAATCCCGTGAAGAAGCTGAAGTACACCCCCGACTATTTCTACACAACCGTCGAGCAGGTGAAGAAGGCTCCCGCCGTAAGCACCAAGGTCACCGCCAGCCCCGCTCCCAGCTACGACCAAGAGTACGTGACTGTGCCCACGCCCAGCGCCTCCGAGGATGTCGACTACTTGGACGTCTACTCGCCCCAGTCGCAGACGCAGAAGCTGAAGAATGGCGACTTTGCCACCCCTCCGCCAACCACGCCCACCTCTCTGCCGCCCCTCGAAGGCATCAGCACGCCACCCCAATCGCCGGGGGAGAAATCCTCGTCAGCTGTCAGCCAGGAGATCAATCATCGAATTGTGACAGCCCCGAATGGAGCCGGCGATTTCAATTGGTCGCACATCGAGGAGACTTTGGCATCAGATTGCAAAGACTACAGCTCCCCGGAGCACTGTGGTGCCGGCTACACCGCGATGCTGCCGCCACTGGAGGCCACAAGCACCGCCACCACCGGGGCACCATCGGTGCCAGTGCCCATGTACCACCACCACCAAACCACCGCCGCCTACCCCGCTTACAGCCACAGTCACAGTCATGGTTATGGCCTGCTCAATGATTACCCTCAGCAGCAGACCCACCAGCAGTACGATGCCTACCCGCAGCAGTACCAACATCAGTGCAGCTACCAGCAACATCCACAGGACCTCTACCATCTGTCTTGAGGTCCGGCGATGCTCAGTTACTCTCTTCCCCAGAGCGGAACCGAAAGCCGTACCGCCACGAAACCGAAGCGCACTTCTCTCGACCATTTGTAGGTGACACGCAAATGACACAGCCGAGAACGAAGCTGCGACGCGATGAGTTGCACAGTAGAGGGCGCACTCCCTACGGTGCCCAGGACATTTTGGGCACAAGGACGAGTGCGCAAGTGCAGAAGGCAGAGGCAAAAGAGGCAGCGCAAACAGAAAAGGAGCCTTGCTGCGCGCGGAACCCAGTGGCTGGCCATGATGGGTTCTCAGCGATCGATTAGCTGCGGCCAAACACAAGCCCAAAACACTCAGCTGGGAGTGATAATGGCCAAGAGACTTGGAGACTGACACACATGTTTTTGTACATATAGTAGTTAAGATATTCCTATCATAGAATTCTATTTATTTAAATATACGAGTAAAGTAAATCGATCGAATTTAAAC | Omitted 831-1016 of mRNA sequence. |
| Dm *eve* CDS submitted to Molecular Instruments | HCR *in situ* | AATGCCTATCCAGTCCGGATAACTCCTTGAACGGCAGCCGCGGCTCGGAGATTCCCGCCGACCCGTCGGTACGCCGCTATCGCACCGCCTTCACCCGTGACCAGCTGGGTCGCTTGGAGAAGGAGTTCTACAAGGAGAACTACGTGTCCCGTCCCCGTCGCTGCGAACTGGCCGCCGTCAGAGGATCGCCGTCGCCTGGCCCTACGCAGCCGTCTACTCCGATCCCGCCTTCGCCGCCTCCATCCTCCAGGCCGCCGCCAACAGCGTGGGCATGCCCTATCCGCCCTACGCCCCCGCTGCTGCCGCCGCTGCTGCCGCCGCCGCTGCCGTGGCCACCAATCCGATGATGGCCACCGGAATGCCCCCGATGGGCATGCCCCAGATGCCCACAATGCAGATGCCCGGACACTCGGGACATGCCGGCCATCCATCGCCCTACGGACAGTACCGCTACACGCCCTACCACATCCCCGCCCGCCCGGCGCCGCCACATCCCGCTGGTCCTCATATGCATCATCCGCACATGATGGGATCCAGCGCAACGGGATCGTCGTACTCCGCCGGTGCCGCCGGCCTTTTGGGCGCTCTGCCCTCCGCCACCTGCTATACCGGACTGGGTGTGGGTGTGCCCAAGACCCAGACGCCGCCGCTGGATCTGCAGTCGTCGTCATCGCCGCACTCCTCCACGCTGTCGCTCTCGCCAGTGGGATCCGATCACGCCAAGGTGTTCGACCGCAGTCCAGTGGCTCAATCCGCTCCATCAGTTCCTGCTCCCGCTCCACTGACCACCACCAGCCCGCTGCCCGCTCCAGGCCTCCTGATGCCCAGTGCCAAGCGGCCTGCCTCCGACATGTCGCCGCCGCCGACGACAACTGTGATTGCGGAGCCCAAGCCGAAGCTCTTCAAGCCCTACAAGACTGAGGCGTAAGCCCGCGATCCACACACACTCTCTCCCCCCCCCCCATGCTCCCCCAAAAGATTGTACAAACTAGTCTTAGTCAGCCTCATCTATTTATTCCCGAAGATTGTACAGATTGTAGAGTAGCTAATTGTAGTCATAATTAAGGCGCAAAATCAAATTAAGAAATAAATGCGAAAATAACATTG | Omitted 1-235 and 411-474 of mRNA sequence. |
