# Supplementary material for: The fushi tarazu zebra element is not required for Drosophila viability or fertility
Source: G3 (Bethesda). 2021 Aug 26;11(11):jkab300. doi: 10.1093/g3journal/jkab300 (PMC8527495; doi:10.1093/g3journal/jkab300)
Supplement: jkab300_Supplementary_Data [file jkab300_supplementary_data.zip › GENETICS-G3-2021-402710-s03.docx]

|  | *ΔZ1c/TM3Sb*  % non-*Sb* (n) | *ΔZ2c/TM3Sb*  % non-*Sb* (n) | *ftzΔZ*  % non-*Sb* (n) | *ΔZ5c/Tm3Sb*  % non-*Sb* (n) | *ΔZ6c/TM3Sb*  % non-*Sb* (n) |
| --- | --- | --- | --- | --- | --- |
| *ΔZ1c/TM3Sb* | 0 (175) |  |  |  |  |
| *ΔZ2c/TM3Sb* | 0 (74) | 0 (354) |  |  |  |
| *ΔZ* | 40 (n=116) | 47 (162) | 100 (133) |  |  |
| *ΔZ5c/TM3Sb* | 12 (n=145) | 30 (105) | 51 (101) | 13 (103) |  |
| *ΔZ6c/TM3Sb* | 30 (n=156) | 22 (194) | 40 (173) | 30 (130) | 0 (110) |

**Supplemental Table 2. Complementation tests suggest second site mutations in *ΔZ1c,2c,5c* and *6c*.**
