# Supplementary material for: The fushi tarazu zebra element is not required for Drosophila viability or fertility
Source: G3 (Bethesda). 2021 Aug 26;11(11):jkab300. doi: 10.1093/g3journal/jkab300 (PMC8527495; doi:10.1093/g3journal/jkab300)
Supplement: jkab300_Supplementary_Data [file jkab300_supplementary_data.zip › GENETICS-G3-2021-402710-s04.docx]

**Supplemental Table 3. Lethal periods of embryos homozygous for *ftz* deletions.**

| Strain | # eggs scored | % hatched^a^ | % pupae^b^ | % adult^c^ | # adults (final %)^d^ |
| --- | --- | --- | --- | --- | --- |
| *w^1118^* | 260 | 81 | 85 | 96 | 159 (61) |
| *ftzΔZp* | 290 | 84 | 81 | 96 | 185 (64) |
| *ftzΔZ* | 250 | 59 | 69 | 79 | 72 (29) |

1. % hatched = (# empty eggshells/# eggs) x100
2. % pupae = (#pupae/# larvae) x100
3. % adult = (# adults/# pupae) x100
4. final % = (# adults/# eggs) x100
