# Supplementary material for: The fushi tarazu zebra element is not required for Drosophila viability or fertility
Source: G3 (Bethesda). 2021 Aug 26;11(11):jkab300. doi: 10.1093/g3journal/jkab300 (PMC8527495; doi:10.1093/g3journal/jkab300)
Supplement: jkab300_Supplementary_Data [file jkab300_supplementary_data.zip › GENETICS-G3-2021-402710-s06.docx]

**Supplemental Table 5. Phenotypes of adults homozygous for *ftz* deletions.**

| Deletion strain | % Normal | % Missing part of A2/A3 | % Missing  all of A2/A3 | % with defects in segments in addition to or other than A2/ A3 |
| --- | --- | --- | --- | --- |
| *ftzΔZp* (n=834) | 50 | 39 | 9 | 2 |
| *ftzΔZ* (n=639) | 2 | 18 | 60 | 20 |
