# Supplementary material for: The fushi tarazu zebra element is not required for Drosophila viability or fertility
Source: G3 (Bethesda). 2021 Aug 26;11(11):jkab300. doi: 10.1093/g3journal/jkab300 (PMC8527495; doi:10.1093/g3journal/jkab300)
Supplement: jkab300_Supplementary_Data [file jkab300_supplementary_data.zip › GENETICS-G3-2021-402710-s07.docx]

**Supplemental_Figure_Legends**

**Figure S1. Polymorphisms in *ftz* found in *nos-Cas9* flies.** Sequences surrounding the zebra element in the *nos-Cas9* line were compared to the *ftz* region of *Drosophila melanogaster* chromosome 3 (AC001653). Features indicated: zebra region (bold, no underline), gRNA sequences (purple, bold and underlined), transcription and translation start sites (green, bold and underlined) and stop codon (dark red, bold and underlined). Several polymorphisms were observed (bright red, bold, and underlined). Three of these occur in the coding sequence, but only one (orange, bold and underlined) results in an amino acid change of a serine to a threonine, which is conservative and unlikely to impact function.

**Figure S2. Example of processing and removal of fluorescent background from 1D analysis.** Plots produced from centroids in central 45-55% of dorsal-ventral axis. Green: *ftz* signal. A) Plot of *ftz* signal from central strip with background present. Note the parabolic curve of background. Black line represents fluorescent intensity threshold; all points below threshold are used in background quadratic model calculations. B) Red line represents the fit parabolic model representing the background. C) Purple: difference between *ftz* with background (green) and background model (red line from B). Black line drawn at 0 to highlight datapoints that became negative (all negative points are between 0 and -1). D) All negative data points converted to 0. This results in the processed dataset used for calculating stripe integrals.

**Figure S3. Partial sequence of the deletion region in *ftzΔZ*p**. PCR products of the region around the zebra element were prepared from single *ftzΔZp* homozygous flies and sequenced to examine the deleted region. Features of interest are indicated with text that is colored, bold and underlined. Indel (pink); portions of the 3’ gRNA remaining (-276 to -261 in diagrams, blue), transcription start site, green (+1 in diagrams) and translation start codon. The 5’ region of the zebra CRE that remains stops 13 bases before the 5’ gRNA (at base -637 in diagrams).

**Figure S4. Partial sequence of the deletion region in *ftzΔZ.*** PCR products of the region around the zebra element were prepared from single *ftzΔZ* homozygous flies and sequenced to examine the deleted region. Features of interest are indicated with text that is colored, bold and underlined: gold, AvrII site; green the transcription start site (+1 in diagrams) and the translation start codon.

**Figure S5. Adult phenotypes in males.** Abdomens from *w^1118^* (A) or *ftzΔZ* (B-C) male flies. Anterior is up. Arrows indicate the darkly pigmented region of segment A3. *ftzΔZ* flies often partially (B) or fully (C) fail to develop portions of segments A2 and A3.

**Figure S6. Perturbations of *slp1* and *en* gene expression in *ftzΔZ* mutants.** Colorimetric *in situ* hybridization to examine *ftz* target gene expression. *slp1* expression in (A) wild type control and (B-C) *ftzΔZ^-/-^* mutants; *en* expression in (D) wild type control and (E-H) *ftzΔZ^-/-^* mutants. A range of phenotypes was observed for *en* staining in *ftzΔZ^-/-^* embryos; E: wild type-like, F: weak stripe 8, G: missing stripe 8, H: multiple abnormalities, including missing stripe 6 and 8 and a weak stripe 10 and 14. Weak stripes conveyed with numbers in parentheses.
