# Supplementary material for: The fushi tarazu zebra element is not required for Drosophila viability or fertility
Source: G3 (Bethesda). 2021 Aug 26;11(11):jkab300. doi: 10.1093/g3journal/jkab300 (PMC8527495; doi:10.1093/g3journal/jkab300)
Supplement: jkab300_Supplementary_Data [file jkab300_supplementary_data.zip › GENETICS-G3-2021-402710-s08.docx]

**Graham, Fischer et al. Fig. S1**

*>Sequenced nosCas9 reads*

TTTTATATCCTACTCTACACCCTTATTGTTAAGTTTACTGCAAAACCAGAAAGGAGGCT

CACATTCACTGGCAATATAGCAATCACAGGACATCGGAGAGATGCGAACTGCAGTCTCT

TCTGTCAAACTGAAAAACGCCATAAATTTTGTGGAACAATGGAATCTGCCAGAGTATGC

GGATGGGGTAAGGGTAGAATTCGCACATGCACACATATGTCAGGAGTATCGATATGTTA

GGGGTGTTCAACGGGGGTGGCTAATAAAATTTATCCAGGCCGCGTAAGCCGATCAGAAA

GTCATGTAAACACAAAAGTGGCTGAGAGATGATTGTTTTTTACCAGTCAATTTAGCTGA

GCTGATTTCAGGTTATACGCGAACCTAAGATGGAAGTAGATTTTTAGATTACCAGAAAT

AACCTTCAGTTTCTAAAAGAATGAGAAATAGTAAATCGT**A**AAGC**A**TGTGGCATTCTTAC

CCCTTACTGAAATGAATGTAACAGGTAGAAGGCAGCAATTGCAGTGCTACAAAGTATAT

ATATTCTTGATCCATATAAGTATAA**T**TATAAGAGCTATAATGTTATAATTAAACATGAA

GATCCTACGCTGT**A**CAAGTTTTGTGCCACAAATTTAACTCAAGATTACC**T**CTCACAAAG

GATACATTACCATTTGGCCATTAAAAATTACAGCATCCATAGACAACCTACTTAAAATT

TATAAAAACTTGAGGGTTATATCACAGAGTTACCGAAAAAAAGCGTAAAAGCTTTATAT

TCTCAACAATATTATGCTATTAAAATATTGCTGGTTTTCTGCTGTTATAGAATCATTTT

TAAAAGTATAACGTAAAAAATAAAATAA**A**CTAGTATTCATTTGAAAA**T**TCAGCGGGCAT

ATAATTTATATCATATTTTTAAAATTTAGGCAAAGGATGTTTGCATAAAGTTTTTACTG

TTTACTAGTCATTTTGGAAGTGCGTTTGTTGGTTTTTAGGCAAATACCGGGCACAGGAG

TGAGTTTGGGAATCGGGAGTTGCGCACTTGCTTGGCCACGAG**GGCAAACAAAAAGCGCA**

**AACACGCGACCCTCGGCCACGCGTATTCCTGATCCCAGGGATCGGACGTAATGTTATCC**

**TTTGGCCGCCCAGTGCCACGAAATAAATTCGGAGGGAAAGGGCATCGGGTTCCGGGAAC**

**AACTGGCAGCCAGTCTTCGGTGTTTTGCGCGCTGGCAAAAATCCAGAGAAATTTTTAGG**

**GAACCATAAACGGGCCGGGGAAAAAGCCTCTGCGCCGAAGGAACGTTTTCAGCAACAGT**

**TTACAGTTTTTATGTCTTTATGATTATTGCAATTAGAGGGAGATCGGCTGAGAGTCGCG**

**CCCTCTCGCTCTGCGCACCTCATAGGTAGGCACCTCATGGCCGTAATTACTGCAGCACC**

**GTCTCAAGGTCGCCGAGTAGGAGAAGCGCGCGGGCGGATAAATCGCGATGATAATGGGC**

**GCGATGGGTAGGTAATAAGCCGCGCAGCAGGTAGGCACCGTACGGATAAAGTTGCCAGG**

**ACCTCGGATAACTTCCCCTCTCCGTGCCTGCAAGGACATTTCGCCGGAGGGGTGGCTGC**

**GAACAGCAGGCGGCAAAGTGTCATGCGCAGGGATATTTATGCGCTATAACGGCGAGCGT**

**GTGCCGAGGGCTCTCTGATTTTGCTATATATGCAGGATCTGCCGCAGGACCAGCTCATT**

**CGCAAACTCACCAGCGTTGCGTGCACATCGCAGAGTTAGAGAAGAAATCTAGCAATACA**

**CATCCGATATGGCCACCACAAACAGCCAGAGCCACTACAGCTACGCCGACAACATGAAC**

ATGTACAACATGTATCACCCCCACAGCCTGCCGCCCACCTACTACGATAA**C**TCAGGCAG

CAATGCCTACTATCAGAACACCTCCAATTATCAGGGCTACTATCCCCAGGAGAGTTACT

CGGAGAGCTGCTACTACTACAACAATCAGGAGCAGGTGACCACCCAGACTGTACCGCCC

GTGCAACCCACCACCCCGCCGCCCAAGGCCACCAAGCGCAAGGCCGAAGATGATGCTGC

TTCCATCATCGCCGCCGTGGAGGAGCGACCCAGCACACTGAGGGCTCTGCTCACCAA**C**C

CCGTGAAGAAGCTGAAGTACACCCCCGACTATTTCTACAC**C**ACCGTCGAGCAGGTGAAG

AAGGCTCCCGCCGTAA**C**CACCAAGGTCACCGCCAGCCCCGCTCCCAGCTACGACCAAGA

GTACGTGACTGTGCCCACGCCCAGCGCCTCCGAGGATGTCGACTACTTGGACGTCTACT

CGCCCCAGTCGCAGACGCAGAAGCTGAAGAATGGCGACTTTGCCACCCCTCCGCCAACC

ACGCCCACCTCTCTGCCGCCCCTCGAAGGCATCAGCACGCCACCCCAATCGCCGGGGGA

GAAATCCTCGTCAGCTGTCAGCCAGGAGATCAATCATCGAATTGTGACAGCCCCGAATG

GAGCCGGCGATTTCAATTGGTCGCACATCGAGGAGACTTTGGCATCAGGTAGGCATCAC

ACACGATTAACAACCCCTAAAAATACACTTTGAAAATATTGAAAATATGTTTTTGTATA

CATTTTTGATATTTTCAAACAATACGCAGTTATAAAACTCATTAGCTAACCCATTTTTT

CTTTGCTTATGCTTACAGATTGCAAAGACTCGAAACGCACCCGTCAGACGTACACCCGC

TACCAGACCCTGGAGCTCGAGAAGGAGTTCCACTTCAATAGATACATCACCCGGCGTCG

TCGCATCGATATCGCCAATGCCCTGAGCCTGAGCGAAAGGCAGATCAAGATCTGGTTCC

AAAACCGACGCATGAAGTCGAAGAAGGATCGCACGCTGGACAGCTCCCCGGAGCACTGT

GGTGCCGGCTACACCGCGATGCTGCCGCCACTGGAGGCCACAAGCACCGCCACCACCGG

GGCACCATCGGTGCCAGTGCCCATGTACCACCACCACCAAACCACCGCCGCCTACCCCG

CTTACAGCCACAGTCACAGTCATGGTTATGGCCTGCTCAATGATTACCCTCAGCAGCAG

ACCCACCAGCAGTACGATGCCTACCCGCAGCAGTACCAACATCAGTGCAGCTACCAGCA

ACATCCACAGGACCTCTACCATCTGTCT**TGA**GGTCCGGCGATGCTCAGTTACTCTCTTC

CCCAGAGCGGAACCGAAAGCCGTACCGCCACGAAACCGAAGCGCACTTCTCTCGACCAT

TTGTAGGTGACACGCAAATGACACAGCCGAGAACGAAGCTGCGACGCGATGAGTTGCAC

AGTAGAGGGCGCACTCCCTACGGTGCCCAGGACATTTTGGGCACAAGGACGAGTGCGCA

AGTGCAGAAGGCAGAGGCAAAAGAGGCAGCGCAAACAGAAAAGGAGCCTGC
