# Supplementary material for: The fushi tarazu zebra element is not required for Drosophila viability or fertility
Source: G3 (Bethesda). 2021 Aug 26;11(11):jkab300. doi: 10.1093/g3journal/jkab300 (PMC8527495; doi:10.1093/g3journal/jkab300)
Supplement: jkab300_Supplementary_Data [file jkab300_supplementary_data.zip › GENETICS-G3-2021-402710-s10.docx]

**Graham, Fischer et al. Fig. S3**

>Sequenced_ftzΔZp_reads

GGAGTATCGATATGTTAGGGGTGTTCAACGGGGGTGGCTAATAAAATTTATCCAGGCCGCGTAAGCCGATCAGAAAGTCATGTAAACACAAAAGTGGCTGAGAGATGATTGTTTTTTACCAGTCAATTTAGCTGAGCTGATTTCAGGTTATACGCGAACCTAAGATGGAAGTAGATTTTTAGATTACCAGAAATAACCTTCAGTTTCTAAAAGAATGAGAAATAGTAAATCGTAAAGCATGTGGCATTCTTACCCCTTACTGAAATGAATGTAACAGGTAGAAGGCAGCAATTGCAGTGCTACAAAGTATATATATTCTTGATCCATATAAGTATAATTATAAGAGCTATAATGTTATAATTAAACATGAAGATCCTACGCTGTACAAGTTTTGTGCCACAAATTTAACTCAAGATTACCTCTCACAAAGGATACATTACCATTTGGCCATTAAAAATTACAGCATCCATAGACAACCTACTTAAAATTTATAAAAACTTGAGGGTTATATCACAGAGTTACCGAAAAAAAGCGTAAAAGCTTTATATTCTCAACAATATTATGCTATTAAAATATTGCTGGTTTTCTGCTGTTATAGAATCATTTTTAAAAGTATAACGTAAAAAATAAAATAAACTAGTATTCATTTGAAAATTCAGCGGGCATATAATTTATATCATATTTTTAAAATTTANGCAAAGGATGTTTGCATAAAGTTTTTACTGTTTACTAGTCATTTTGGAAGTGCGTTTGTTGGTTTTTANGCAAATACCGGGCACAGGAGTGAGTTTGGGAATCGGGAGTTGCGCACTTGCTTGGCCACGAGGGCAAACAAAAAGCGCAAACACGCGA**ACAAACAGGTAGGCACCGTA**CGGATAAAGTTGCCAGGACCTCGGATAACTTCCCCTCTCCGTGCCTGCAAGGACATTTCGCCGGAGGGGTGGCTGCGAACAGCAGGCGGCAAAGTGTCATGCGCAGGGATATTTATGCGCTATAACGGCGAGCGTGTGCCGAGGGCTCTCTGATTTTGCTATATATGCAGGATCTGCCGCAGGACCAGCTC**A**TTCGCAAACTCACCAGCGTTGCGTGCACATCGCAGAGTTAGAGAAGAAATCTAGCAATACACATCCGAT**ATG**GCCACCACAAACAGCCAGAGCCACTACAGCTACGCCGACAACATGAACATGTACAACATGTATCACCCCCACAGCCTGCCGCCCACCTACTACGATAACTCAGGCAGCAATGCCTACTATCAGAACACCTCCAATTATCAGGGCTACTATCCCCAGGAGAGTTACTCGGAGAGCTGCTACTACTACAACAATCAGGAGCAGGTGACCACCCAGACTGTACCGCCCGTGCAACCCACCACCCCGCCGCCCAAGGCCACCAAGCGCAAGGCCGAAGATGATGCTGCTTCCATCATCGCCGCCGTGGAGGAGCGACCCAGCACACTGAGGGCTCTGCTCACCAACCCCGTGAAGAAGCTGAAGTACACCCCCGACTATTTCTACACCACCGTCGAGCAGGTGAAGAAGGCTCCCGCCGTAACCACCAAGGTCACCGCCAGCCCCGCTCCCAGCTACGACCAAGAGTACGTGACTGTGCCCACGCCCAGCGCCTCCGAGGATGTCGACTACTTGGACGTCTACTCGCCCCAGTCGCAGACGCAGAAGCTGAAGAATGGCGACTTTGCCACCCCTCCGCCAACCACGCCCACCTCTCTGCCGCCCCTCGAAGGCATCAGCACGCCACCCCAATCGCCGGGGGAGAAATCCTCGTCAGCTGTCAGCCAGGAGATCAATCATCGAATTGTGACAGCCCCGAATGGAGCCGGCGATTTCAATTGGTCGCACATCGAGGAGACTTTGGCATCAGGTAGGCATCACACACGATTAACAACCCCTAAAAATACACTTTGAAAATATTGAAAATATGTTTTTGTATACATTTTTGATATTTTCAAACAATACGCAGTTATAAAACTCATTAGCTAACCCATTTTTTCTTTGCTTATGCTTACAGATTGCAAAGACTCGAAACGCACCCGTCAGACGTACACCCGCTACCAGACCCTGGAGCTCGAGAAGGAGTTCCACTTCAATAGATACATCACCCGGCGTCGTCGCATCGATATCGCCAATGCCC
